# Protein Modifications as Potential Biomarkers in Breast Cancer

**DOI:** 10.4137/bmi.s2557

**Published:** 2009-11-30

**Authors:** Hongjun Jin, Richard C. Zangar

**Affiliations:** Cell Biology and Biochemistry Group, Fundamental and Computational Sciences Directorate, Pacific Northwest National Laboratory, PO Box 999, 902 Battelle Blvd, Richland, WA 99352

**Keywords:** PTMs, post-translational modifications, breast cancer, biomarkers

## Abstract

A variety of post-translational protein modifications (PTMs) are known to be altered as a result of cancer development. Thus, these PTMs are potentially useful biomarkers for breast cancer. Mass spectrometry, antibody microarrays and immunohistochemistry techniques have shown promise for identifying changes in PTMs. In this review, we summarize the current literature on PTMs identified in the plasma and tumor tissue of breast-cancer patients or in breast cell lines. We also discuss some of the analytical techniques currently being used to evaluate PTMs.

## Introduction

Viable cells maintain membrane integrity, cytoskeleton morphology and proliferation status based on changes in protein structure and function. The complexity of regulation of so many different biomolecules goes beyond the “central dogma” of biochemistry, which implies that one gene encodes for one protein. This complexity of regulation not only results from variable mRNA splicing and DNA transcription, such that one gene can produce many mRNA and protein sequences, but also because one protein sequence can have multiple functions as a result of covalent modifications after synthesis. These post-translational modifications (PTMs) include phosphorylation, methylation, glycosylation, acylation, oxidation and ubiquitinylation. During cancer progression, many PTMs contribute to abnormal cellular proliferation, adhesion characteristics and morphology. 1–4 In breast cancer, recent studies suggest that PTM profiles can be used as “biochemical foot-prints” for tracking and verifying the function and activity of key cellular signaling pathways.^5^–^7^ This conclusion suggests that, for early detection, PTMs may be useful biomarkers.

Breast cancer is the second most common type of cancer (after lung cancer), and the fifth most common cause of cancer death. According to the American Cancer Society, in 2008, an estimated 182,460 new cases of invasive breast cancer were diagnosed among US women. Approximately 40,480 of these women are expected to die from this disease (http://www.cancer.org/downloads/STT/2008CAFFfinalsecured.pdf). Like many other cancers, breast cancer is the result of multiple environmental and hereditary factors. Although risk factors such as lesions to DNA, failure of immune surveillance, abnormal growth factor signaling, and inherited or somatic genetic defects (e.g. in p53, BRCA1, BRCA2 genes) are associated with breast cancer development, the cause of any individual breast cancer case is typically unknown. As many studies have suggested, changes in gene expression levels for breast cancer may not fully reflect the true state of cancer progression or development.^5^,^8^,^9^ This conclusion suggests that many of the differences between normal and cancer tissue may be caused by PTMs.^1^,^3^,^5^,^6^,^8^–^10^

This review mainly focuses on the most recent publications on PTMs (especially oxidation and glycosylation) discovered in blood or tissue from breast cancer patients or from breast cancer cell lines. For more general reviews of PTMs, see prior reviews.^1^–^4^,^7^,^11^

## Enzymatic PTMs

Covalent modification of one or more amino acids of a given protein can dramatically alter the biological function of that protein. The likelihood that a particular reactive protein residue will undergo a modification reaction is influenced by the spatial orientation of that amino acid residue(s) in the protein, and is influenced by the adjacent amino acids, which can alter reactivity of the susceptible amino acid by influencing its electrophilic nature. Specific enzymes commonly catalyze these reactions. For example phosphorylation (phosphokinase), methylation (methylase), acetylation (acetyltransferase), and glycosylation (glycosyltransferases) are PTMs that are mediated by the indicated enzyme.^1^–^4^,^7^,^11^ Many PTMs also result from spontaneous reaction of susceptible residues with certain reactive chemical agents. For example, glycation (commonly called advanced glycation end product, or AGE) is the result of an activated sugar molecule, such as fructose or glucose, bonding to a protein without direct enzymatic involvement.^12^ For other PTMs, although enzymes may play an important role in producing the reactive molecule that results in the protein modification, the covalent modification occurs spontaneously without enzymatic activity. For the purposes of this review, we define these PTMs as non-enzymatic if an enzyme is not required for the actual protein modification. For example, peroxynitrite directly reacts with proteins to form nitrotyrosine.^13^–^15^ Although peroxynitrite formation appears to require enzymatic production of reactive precursors, its binding to proteins is non-enzymatic, and therefore we consider nitrotyrosine to be a non-enzymatic PTM.

## Phosphorylation

Phosphorylation is well recognized as a key regulator of enzyme activity. As the extensive research in protein phosphorylation has been carefully reviewed by others,^16^–^18^ we only briefly cover this topic here. Abnormal phosphorylation of defined signal transduction pathways can alter the growth properties of breast tumors. With the use of sequence-specific antibodies against phosphorylation sites, analysis of protein phosphorylation profiles allows one to determine the activation status of signaling pathways, which can provide valuable prognostic insights.^19^–^21^ Atsriku et al undertook a systematic mapping of PTMs in the human estrogen receptor alpha (ER-α) in the MCF7 breast cancer cell line. They applied HPLC-ESI and MALDI-MS techniques to identify the phosphorylation sites on the estrogen receptors in these cells.^22^ Several novel phosphorylated serine residues were identified. The use of both HPLC-ESI and MALDI gave higher sequence coverage than either approach alone. Nine phosphorylated serine residues were identified, three of which were previously unreported.^22^

## Acetylation

Histone acetyltransferases and histone deacetylases modify histones by adding or removing an acetyl group from the ∑-amino group of lysines within a conserved lysine motif. Histone acetylation results in changes in chromatin structure in response to specific endocrine signaling in several cancers, including breast cancer. Recent studies found that acetylation of the ER is mediated by histone acetylases.^23^–^25^ The acetylation of ER-α alters its function in estrogen-dependent signaling.^23^,^24^ The regulation of ER by deacetylation provides a direct link between intracellular metabolism and hormone signaling.^25^,^26^ Wang et al^27^ showed that the acetylation of ER-α alters its function *in vitro* and *in vivo*. These researchers also found that p300 selectively and directly acetylated the ER-α at lysine residues within the ER-α hinge/ligand-binding domain. Substitution of these residues with charged or polar residues dramatically enhanced ER-α hormone sensitivity without affecting induction by MAPK signaling.^27^ These results suggest that ER-α acetylation normally suppresses ligand sensitivity.

## Glycosylation

Cancer cells commonly have unusually high levels of certain types of tumor-associated glycans.^28^ Specific antibodies are available for these unusual carbohydrate residues, and there is considerable evidence that these glycans are increased in breast cancer.^12^,^29^,^30^ Differences in protein glycosylation commonly result from differences in the activities and subcellular (primarily Golgi and endoplasmic reticulum) localization of glycosyltransferases that determine the amounts of specific glycans.^31^–^33^ Several glycosylation modifications, such as TF and Tn antigens, certain Lewis antigens and Globe H (summarized in Table 1), are commonly associated with a variety of different cancers, including breast cancer. Glycoprotein analysis by mass spectrometry (MS) of biological samples, such as blood serum, is hampered by glycan complexity and the low concentrations of the potentially informative glycopeptides and proteins. Most MS-based studies have limited their analysis to glycosylation residues after cleavage of the glycans from the proteins. As such, these studies can identify global changes in glycosylation, but do not provide information on which proteins are modified.

Changes in glycosylation for cancer cells include both reductions and increases in naturally occurring glycans, as well as increases in glycans primarily restricted to embryonic tissues.^34^,^35^ One of the most common changes is an increase in the side branching of N-linked glycans.^36^ This increased branching is often attributed to increased activity of N-acetylglucosaminyltransferase V (GlcNAc-T V, also known as MGAT5; the enzyme that leads to β1,6GlcNAc branching).^37^–^40^ The increased branching creates additional sites for terminal sialic acid residues, which, in combination with up-regulation of sialyltransferases, leads to an increase in global sialylation.^41^

In addition to changes in glycan core structures, altered terminal structures are commonly associated with malignant breast cancer.^42^–^47^ Glycosyltransferses (e.g. sialyltransferases and fucosyltransferases) involved in adding terminating residues to glycans tend to be over-expressed in breast cancer tissue.^29^,^30^,^48^–^66^ The increase in activity of these glycosyltransferases, in turn, leads to an increase of certain terminal glycans. Glycan residues commonly found on transformed cells include sialyl Lewis x, sialyl Tn, Globo H, Lewis y and polysialic acid. Many of these glycans are observed in malignant breast tissues (summarized in Table 1).

## Non-Enzymatic PTMs

### Oxidation

It has been hypothesized that cancer development is a process that is similar to “wounds that never heal”.^67^–^70^ Various studies have suggested that inflammation, which increases oxidative stress, is associated with cancer development or metastasis.^67^,^71^–^74^ Both mouse models and human-pathology studies suggest that there is a strong immune response in the early stages of breast cancer that disappears in more advanced disease.^72^,^75^ Consistent with this observation, tumor levels of nitrotyrosine (nTyr), which are believed to be indicative of NO and superoxide levels, have been reported to be increased in the early breast cancer, but not in more advanced disease.^72^,^75^ The NO and superoxide may be produced by activated macrophages. Therefore, localized oxidative stress associated with the immune response to breast cancer might result in modifications of proteins secreted by the breast cancer cells that could be used to detect early disease. Reactive oxygen species (ROS) also regulate the synthesis and secretion of many receptor ligands (e.g. growth factors and chemokines).^76^–^78^ These factors regulate important processes in epithelial cancers, including the ligand-dependent activation of the proliferation (MAPK/ Erk) and anti-apoptosis (PI3K/Akt) pathways.^79^–^81^ Therefore, proteins modified by ROS may be useful biomarkers that can provide insight into molecular processes occurring in tumors. The oxidative stress associated with the immune response results in protein modifications that may be useful in detecting early breast cancer.

An increase in 4-hydroxynonenal (4-HNE) adducts has also been reported in early breast cancer.^82^,^83^ 4-HNE is a non-enzymatic byproduct of lipid peroxides. 71 Lipid peroxidation and HNE adducts may result from oxidative stress associated with the immune response.^71^,^72^ There is, however, also evidence that the intracellular redox environment is altered in breast cancer,^84^–^87^ potentially leading to a variety of PTMs. Notably, levels of reduced glutathione (GSH) have been reported to be altered in breast cancer tissue.^88^–^91^ The literature on oxidative modifications (i.e. on 4-HNE, nTyr and GSH adducts) is summarized in Table 2. Each of these oxidative modifications represents a different cellular process; that is, 4-HNE adducts are a byproduct of lipid peroxidation, nTyr commonly results from an increase in NO (produced by either macrophages or breast epithelial cells) and GSH protein adducts can be indicative of intracellular oxidative stress, especially in the endoplasmic reticulum.^71^,^72^,^88^–^92^

## Advanced Glycation End

Oxidative and carbonyl stress may contribute to the progression of cancer; on the other hand, these modifications may have some antiproliferative effects. Tesarova et al^12^ reported that serum levels of AGEs, carboxymethyllysine and advanced oxidation protein products (AOPP) in 86 patients with breast cancer and in 14 healthy age-matched control women could be subdivided based on the clinical stage, histological grading, and expression of hormone and Her2 receptors. Breast cancer patients had higher serum concentrations of AGEs even in the early stages of this disease; patients with advanced breast cancer (stages III and IV) had significantly higher AGE levels, not only compared to controls, but also compared to stages I and II breast cancer cases.^12^,^70^,^93^ Serum levels of AOPP were higher in patients having only weakly positive expression of Her2 compared to controls and in patients having the highest Her2 expression.^12^ These authors concluded that breast cancer patients had an early increase of AGEs (a marker of the carbonyl stress) followed by further increase of AGEs and elevation of AOPP (a marker of oxidative stress) in more advanced disease. As the clinical significance of these observations is currently uncertain, further studies are needed to validate these results in terms of the usefulness of AGE in the early detection of breast cancer.

## Methods for PTM Discovery and Analysis

### Mass-spectrometry-based proteomics

Given the complexity and low abundance of the PTM samples, PTM analysis is still an analytical challenge. Various mass spectrometry (MS) technologies, including ion trap, time-of-flight (TOF), Orbitrap, and Fourier transform ion cyclotron resonance (FTICR), as well as hybrid configurations coupled with MALDI have been used for PTM detection in breast cancer studies (Table 1). Recent applications commonly include multi-stage separation, purification and enrichment of the PTM-containing peptides or proteins.^7^,^94^–^98^ The most frequently used proteomics approaches for PTM analysis may be MALDI TOF, electrospray ionization tandem MS that uses LTQ–Orbitrap instrumentation, and surface-enhanced laser desorption/ionization (SELDI)-MS.^99^–^104^ For the MALDI and SELDI approaches, the profile of peak intensities in case and control samples are typically compared with the goal of defining a pattern that can segregate the sample types. Many analyses of PTMs in serum samples from breast cancer patients have been recently reported (Tables 1–3).

Most PTMs are present at low levels in cells and tissues, and are therefore difficult to detect by MS. For this reason, modification-specific analytical strategies that are designed to improve sensitivity and specificity have been employed to enrich and concentrate a specific class of PTM in complex biological samples. PTM peptide enrichment can employ either affinity^105^ or chemical methods.^12^,^106^–^110^ During the MS analysis, multi-stage MS techniques that further fragment suspected PTM peptides^111^ can improved confidence in peptide identification. Identification of PTMs commonly requires specialized bioinformatics tools, the validation of results by replicate analyses^42^–^47^ and follow-up biological experiments.^112^ Such PTM-specific methods can be combined with semi-quantitative techniques, including stable-isotope labeling and peptide-intensity profiling. PTM-targeted methods have also been combined with subcellular fractionation to obtain biological insights about in the roles of specific organelles.^113^–^117^

## ELISA Microarray

The microarray sandwich ELISA is an exceptionally sensitive analytical technique that can accurately measure individual protein concentrations down to the low or sub-pg/ml range.^115^,^117^–^120^ Adapted from the conventional sandwich ELISA, the ELISA microarray commonly uses complementary pairs of capture and detection antibodies (or, for glycan analysis, lectins) to measure trace antigens in complex biological fluids. The microarray technique is also suited for targeted discovery research because of its ability to simultaneously conduct multiple assays. At the same time, this multiplex analysis requires very little sample (20 μl, or less, of diluted sample per multiplexed analysis, after at least a 5-fold dilution), thereby allowing the screening of many PTMs using very small sample volumes. Even so, there are several challenges for ELISA microarray analysis. One challenge is the need for highly specific antibodies. There is limited commercial availability of good antibodies for many PTMs. Classical strategies of antibody generation by animal immunization may not result in high-quality antibodies for the targeted PTM. The second challenge is the potential for cross reactivity with nonspecific antigens.

## Immunohistochemistry

Immunohistochemistry (IHC) has been widely used for evaluating PTMs in breast cancer.^113^,^116^,^121^ To identify PTMs as potential tumor markers, IHC offers a rapid method for comparing PTM levels in cancer tissue and adjacent normal tissue. Altered expression and PTM of several proteins using immunoblot analysis and IHC have been reported by several research groups (Tables 1–3). For example, modification of the beta subunit of prolyl-4-hydroxylase and of annexin A2 in tumor tissues was confirmed by immunoblot and immunohistochemistry.^122^ The determination of nitrotyrosine levels by IHC of breast cancer carcinoma tissue has been reported.^75^ A drawback of IHC in PTM analysis is the difficulty in quantifying the results.

## Conclusion

Plasma-, tissue- or cell-based studies for PTM biomarkers in breast cancer have provided promising data. Several PTMs can only be readily detected in breast cancer tissue but not in normal breast. In particular, glycosylation and oxidative modifications appear to have potential as biomarkers. These results suggest that levels of certain PTMs may be indicative of breast cancer progression or development, although the data on which proteins are actually modified is still very limited. Once this deficit is addressed, we conclude that the post-translational modifications on specific proteins may be useful as biomarkers for breast cancer.

## Figures and Tables

**Table 1:** Summary of recent glycosylation PTMs in breast cancer studies.

Cancer glycan	Targets	Methods	References
Sialyl Lewis a (sLe^a^)	E-cadherin, CatD	IHC	45,123
Sialyl Lewis x (sLe^x^)	Serum protein	IHC	42–47
	C2GnT1	MS	
	Mucin glcNac beta1–6 galNac alpha core		
Sialyl Tn (sTn)	St6GalNac-I sialyltransferase	IHC, FACS, MS	48–53
Thomsen-Friedenreich (TF)	Muc1 secretory/shed	MS	29,54–59
	Serum proteins	IHC	
Lewis y (Le^y^)	Not known	IHC	60–62
Globo H	Fucosyl transferase 1,2	Microarray	30,63–66
Polysialic acid	Alpha 2,8-polysialylated glycoprotein	IHC	124
Fucosyl GM1	Blood group-related antigens	IHC	125
GM2	Malignancy tissue	IHC	28

**Abbreviations:** MS, mass spectroscopy; IHC, immunohistochemistry; GlcNAc, N-acetylglucosamine; GM, genetically modified sugar; CatD, Cathepsin D.

**Table 2:** Summary of recent oxidation PTMs in breast cancer studies.

Oxidation PTMs	Targets	Methods	References
Total Oxidation	Blood, NAF proteins; Cytochrome P450	MS, IHC	12,85,106–110,126,127
Nitrotyrosine	VEGF-C	IHC	128,129
Nitrotyrosine	NF-κB	Gel shift	130
Nitrotyrosine	CXCR4, hyaluronan Serum proteins	IHC	72,75,114,128,129
Thiobarbituric acid reactive substances (TBARS)	Lipid	HPLC	88,89,131
Conjugated dienes (CD)	Serum proteins	HPLC	88,89
Glutathione (GSH)	Serum proteins	Enzymatic measurements	88,90,91,132,133
4-hydroxy-2-nonenal (4HNE)	p53	MS, Immunoassay	82,83
3-Chloro Tyrosine*	Chronic rhinosinusitis	MS	134
3-Bromo Tyrosine*	Chronic rhinosinusitis	MS	134
Advanced Glycation end (AGE) product	sRAGE glyoxalase I	IHC	12,70,93

* Not from breast cancer studies.

**Table 3:** Summary of Enzymatic PTMs biomarkers in breast cancer research.

PTM	Targets	Methods	References
Phosphorylation	Nuclear receptor	Mutagenesis	135
Phosphorylation	Estrogen receptor (ER)	Mutagenesis	136
Acetylation	H4K16, histone acetyltransferase human MOF (hMOF)	mRNA profile	137–140
Acetylation	ER-α	Mutagenesis	27
Deacetylation	Histone deacetylase (HDAC)3 histone H4	Small interfering RNA	131
Glycosylation	N-linked glycomics, serum proteins	MADLI MS	141
Glycosylation	Serum glycan	MALDI mass spectrometry (MS)-based glycomic profile	112
Glycosylation	O-glycosylation TGF beta 1	Using 2-De and MALDI-TOF-MS	142
Glycosylation	Free glycan species from serum	MALDI-FT-ICR MS	111
Glycosylation	Glycoproteins from the sera	Multilectin affinity chromatography (MLAC)	105

## Abbreviations

AGEs: advanced glycation end products
BRCA: breast-cancer susceptibility gene
CD: conjugated dienes
DCIS: ductal carcinoma in situ
ELISA: enzyme-linked immunosorbent assay
ER: estrogen receptor
4-HNE: 4-hydroxynonenal
HPLC-ESI: high-performance liquid chromatography-electrospray ionization-mass spectroscopy
IHC: immunohistochemistry
GlcNAc: N-acetylglucosamine
MGAT5: N-acetylglucosaminyltransferase V
GSH: glutathione
GSTP1: glutathione S-transferase P1
MAPK/ERK: mitogen-activated protein kinase/extracellular signal-regulated kinase
LCIS: lobular carcinoma in situ
MALDI: matrix-assisted laser desorption ionization-mass spectroscopy
NO: nitric oxide
nTyr: 3-nitrotyrosine
RASSF1A: RAS association family 1 gene
PI3K/Akt: phosphoinositide-3 kinase/protein kinase B
PTMs: post-translational modifications
ProMAT: Protein Microarray Analysis Tool
ROS: reactive oxygen species
sLe^a^: sialyl lewis a
sLe^x^: sialyl lewis x
sTn: sialyl Tn
Le^y^: Lewis y
SELDI: surface-enhanced laser desorption/ionization
TOF: time-of-flight
MS: mass spectroscopy
TBARS: thiobarbituric acid reactive substances
TF: Thomsen-Friedenreich
